# Structural and functional interactions between the EF hand domain and S2–S3 loop in the type-1 ryanodine receptor ion channel

**DOI:** 10.1016/j.jbc.2023.105606

**Published:** 2023-12-28

**Authors:** Venkat R. Chirasani, Millar Elferdink, MacKenzie Kral, Jordan S. Carter, Savannah Heitmann, Gerhard Meissner, Naohiro Yamaguchi

**Affiliations:** 1 Department of Biochemistry and Biophysics, University of North Carolina at Chapel Hill, Chapel Hill, North Carolina, USA; 2 R.L. Juliano Structural Bioinformatics Core, University of North Carolina at Chapel Hill, Chapel Hill, North Carolina, USA; 3 Department of Regenerative Medicine and Cell Biology, Medical University of South Carolina, Charleston, South Carolina, USA; 4 Cardiac Signaling Center of University of South Carolina, Medical University of South Carolina and Clemson University, Charleston, South Carolina, USA; 5 College of Charleston Honors College, Charleston, South Carolina, USA

**Keywords:** ryanodine receptor, Ca^2+^-dependent regulation, hydrogen bonding, mutation analysis, skeletal muscle disorders

## Abstract

Previous cryo-electron micrographs suggested that the skeletal muscle Ca^2+^ release channel, ryanodine receptor (RyR)1, is regulated by intricate interactions between the EF hand Ca^2+^ binding domain and the cytosolic loop (S2–S3 loop). However, the precise molecular details of these interactions and functional consequences of the interactions remain elusive. Here, we used molecular dynamics simulations to explore the specific amino acid pairs involved in hydrogen bond interactions within the EF hand—S2–S3 loop interface. Our simulations unveiled two key interactions: (1) K4101 (EF hand) with D4730 (S2–S3 loop) and (2) E4075, Q4078, and D4079 (EF hand) with R4736 (S2–S3 loop). To probe the functional significance of these interactions, we constructed mutant RyR1 complementary DNAs and expressed them in HEK293 cells for [^3^H]ryanodine binding assays. Our results demonstrated that mutations in the EF hand, specifically K4101E and K4101M, resulted in reduced affinities for Ca^2+^/Mg^2+^-dependent inhibitions. Interestingly, the K4101E mutation increased the affinity for Ca^2+^-dependent activation. Conversely, mutations in the S2–S3 loop, D4730K and D4730N, did not significantly change the affinities for Ca^2+^/Mg^2+^-dependent inhibitions. Our previous finding that skeletal disease-associated RyR1 mutations, R4736Q and R4736W, impaired Ca^2+^-dependent inhibition, is consistent with the current results. *In silico* mutagenesis analysis aligned with our functional data, indicating altered hydrogen bonding patterns upon mutations. Taken together, our findings emphasize the critical role of the EF hand-S2–S3 loop interaction in Ca^2+^/Mg^2+^-dependent inhibition of RyR1 and provide insights into potential therapeutic strategies targeting this domain interaction for the treatment of skeletal myopathies.

Ryanodine receptor (RyR) calcium release channels are critical components of muscle contraction, responsible for releasing Ca^2+^ from the sarcoplasmic reticulum during action potentials (1, 2). Two major isoforms, RyR1 in skeletal muscle and RyR2 in cardiac muscle, are homotetrameric structures consisting of approximately 5000 amino acid subunits, with the majority of their structure located on the cytosolic side (3). These ion channels are regulated by a complex interplay of physiological factors (Ca^2+^, Mg^2+^, and ATP) and pharmacological molecules (caffeine, 4-CmC, and suramin), indicating the presence of multiple regulatory domains within their large cytoplasmic region. Understanding the intricate regulatory mechanisms of RyRs is crucial for unraveling the intricacies of muscle physiology and associated disorders. One intriguing aspect of RyR regulation is the differential sensitivity to inhibitory Ca^2+^ concentrations between RyR1 and RyR2. While RyR1 is inhibited at Ca^2+^ concentrations above 100 μM, RyR2 requires approximately 10-fold higher Ca^2+^ concentrations for inhibition (4, 5). Our previous studies with RyR1/RyR2 chimeric proteins demonstrated that this difference in inhibitory Ca^2+^ affinity was attributed, at least in part, to the EF hand low affinity Ca^2+^ binding domain, which spans amino acids 4065 to 4298 in rabbit RyR1 and amino acids 4020 to 4250 in RyR2 (6). Another region of interest is the cytosolic loop between the second and third transmembrane segments (S2–S3 loop). Mutations within this loop have been shown to significantly reduce Ca^2+^-dependent inhibition of RyR1 (7), suggesting its involvement in the regulatory mechanisms. To gain insights into the potential interplay between the EF hand domain and the S2–S3 loop, it is crucial to explore their structural proximity and possible interactions.

Recent advances in cryo-EM have provided near-atomic resolution structures of RyR1, shedding light on its architecture and organization. These studies have revealed that the EF hand domain and the S2–S3 loop are in close proximity, suggesting the possibility of domain interactions between them (8). Building upon this structural information, we hypothesized that the interaction between the EF hand and S2–S3 loop contributes to the Ca^2+^-dependent inhibition of RyR1 channel activity. To investigate this hypothesis, we identified the potential amino acid residues involved in hydrogen bond interactions between the EF hand and S2–S3 loop of RyR1 using molecular dynamics simulations. Subsequently, we introduced point mutations at the predicted interaction sites in RyR1 and evaluated the impact on RyR1 ion channel activities.

Through this comprehensive approach, we aimed to unravel the molecular mechanisms underlying the Ca^2+^-dependent regulation of RyR1. By elucidating the specific domain interactions between the EF hand and S2–S3 loop, we seek to enhance our understanding of RyR1 channel regulation and its implications in muscle physiology and pathophysiology. Our study may have implications for the development of novel therapeutic strategies targeting RyR1 dysfunction in the muscle-related disorders.

## Results

### Amino acid residues for domain interactions between EF hand and S2–S3 loop of RyR1

**Figure 1:**
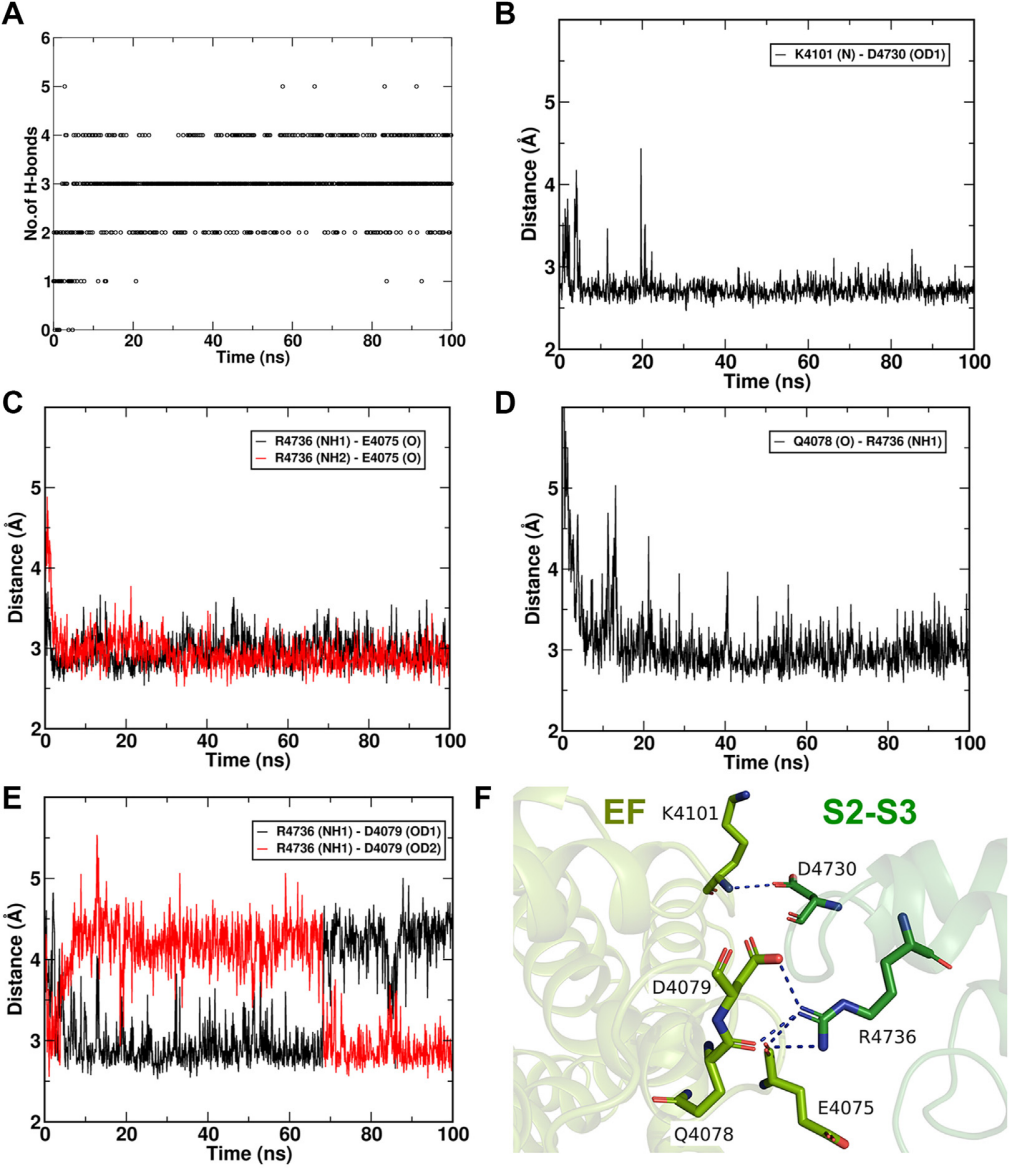
**Molecular dynamics simulations of RyR1 structure to identify hydrogen bond interactions between EF-hand and S2–S3 domains.** The membrane-embedded open state of RyR1 (PDB: 5TAL) was simulated using GROMACS to investigate potential hydrogen bond interactions between the EF hand and S2–S3 loop domains. *A*, our molecular dynamics simulation revealed up to six hydrogen bond interactions, with five hydrogen bonds being the maximum observed in a single trajectory frame. *B*, the EF hand residue K4101 exhibited proximity to D4730 in the S2–S3 loop. *C*–*E*, the S2–S3 loop residue R4736 interacted with (*C*) E4075, (*D*) Q4078, and (*E*) D4079 in the EF hand domain. *F*, a snapshot of the domain interface between the EF hand and S2–S3 loop is shown, highlighting the presence of five hydrogen bonds except for the R4736 (NH1)-D4079 (OD1) interaction. RyR, ryanodine receptor; PDB, Protein Data Bank.

In high-resolution cryo-EM structural analysis of a homo-tetrameric RyR1, it was found that the EF hand domain in one subunit is in proximity with the S2–S3 loop in the neighboring subunit (8). Since the EF hand region was previously identified as a low affinity Ca^2+^ binding site (9, 10), we hypothesized that interaction between the EF hand and S2–S3 loop is important for low affinity Ca^2+^-dependent inhibition of RyR1. We performed a computer simulation based on the published RyR1 structure (Protein Data Bank (PDB) accession 5TAL) (11) and found up to six possible hydrogen bond interactions between the two domains (1): K4101 in the EF hand interacts with D4730 in the S2–S3 loop, and (2) E4075, Q4078, and D4079 in the EF hand interact with R4736 in the S2–S3 loop (Fig. 1). We reported previously that malignant hyperthermia associated R4736W- and R4736Q-RyR1 mutations impaired low affinity Ca^2+^-dependent inhibition (7), consistent with hydrogen bond formation predicted by our computer simulation. Recently, Nayak and Samso (12) reported that distances between K4101 and D4730 as well as E4075 and R4736 are dynamically changed in their cryo-EM structures of closed, open, and inactivated RyR1 as determined in the presence of different Ca^2+^ concentrations, which matches well with our computer simulations. In this article, we further characterize the role of K4101 and D4730 on RyR1 inhibition using site-directed mutagenesis. We constructed five mutant RyR1s. All mutant RyR1s retained Ca^2+^-dependent [^3^H]ryanodine binding, and the gains of Ca^2+^-dependent activity (see Experimental procedures) of the mutants are comparable to that of WT-RyR1 (Table 1).

**Table 1:** Gain of Ca^2+^-dependent activities of WT and mutant RyRs

RyR1 mutation	B_peak_/B_max_ (%)
WT-RyR1	26 ± 12 (8)
K4101A	38 ± 10 (5)
K4101E	22 ± 11 (9)
K4101M	33 ± 11 (7)
D4730K	28 ± 4 (6)
D4730N	43 ± 17 (5)

**Figure 2:**
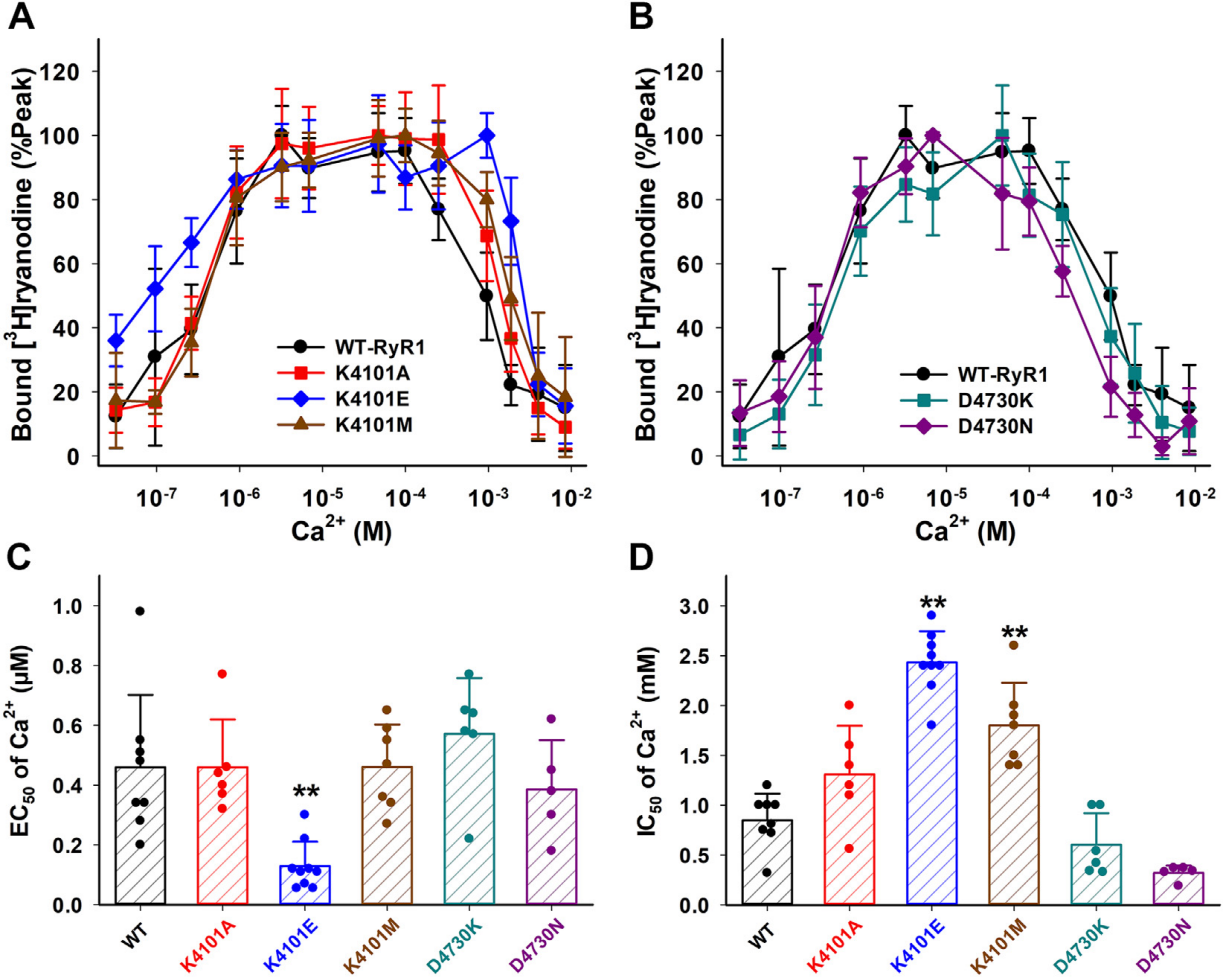
**Ca**^**2+**^**dependent activity changes of WT and mutant RyR1s.** Activities of WT and mutant RyR1s were determined in the presence of various concentration of Ca^2+^ by [^3^H]ryanodine binding methods. *A* and *B*, Ca^2+^ dependent of bound [^3^H]ryanodine of K4101- and D4730-RyR1 mutants are shown together with WT-RyR1 in Panel *A* and *B*, respectively. *C* and *D*, half maximal Ca^2+^ concentrations for Ca^2+^ activation (EC_50_) and inhibition (IC_50_) are shown in Panel *C* and *D*, respectively. Data are mean ± SD (n=5–9). ∗*p* < 0.05 and ∗∗*p* < 0.01 compared with WT-RyR1 by one-way ANOVA followed by Tukey’s test of six different genotypes of RyR1. RyR, ryanodine receptor.

Activities of WT and mutant RyR1s were obtained by normalizing the peak values of Ca^2+^ dependent activity in Figure 2 to the B_max_ values. Data are mean ± SD of the number of experiments indicated in parentheses. None of the mutants are significantly different from WT-RyR1 by one-way ANOVA followed by Tukey’s test.

### Calcium dependent regulation of mutant RyR1s

To gain insights into the roles of a positive charge of RyR1-K4101 for hydrogen bond interaction between the EF hand and S2–S3 domain, we replaced it with glutamic acid (negatively charged), methionine (uncharged with similar amino acid volume), or alanine (uncharged with smaller volume). [^3^H]Ryanodine binding to the recombinant mutant RyR1s determined the apparent Ca^2+^ affinities for ion channel activation and inhibition (Fig. 2). As shown in Figure 2*A*, K4101E-RyR1 exhibited a largely impaired Ca^2+^-dependent inhibition as compared with WT-RyR1, while K4101M-RyR1 showed a modest decrease in inhibitory effect. Ca^2+^-dependent inhibition of K4101A-RyR1 was not significantly different from that of WT-RyR1. Half maximal concentrations of inhibitory Ca^2+^ (IC_50_) were 0.85 ± 0.27, 1.3 ± 0.5, 2.4 ± 0.3, and 1.8 ± 0.4 (in mM) for WT-, K4101A-, K4101E-, K4101M-RyR1, respectively (Fig. 2*D*). On the other hand, mutations at D4730 in the S2–S3 domain did not cause impairments in Ca^2+^-dependent inhibition (Fig. 2*B*). We replaced the acidic amino acid D4730 with lysine (basic) or asparagine (uncharged). The IC_50_ of inhibitory Ca^2+^ for D4730K and D4730N were 0.61 ± 0.32 and 0.32 ± 0.07 mM, respectively. The average IC_50_ values observed for D4730 mutants were slightly lower than those for WT-RyR1; however, these variances did not reach statistical significance (Fig. 2*D*). Additionally, the statistical analysis of IC_50_ values in Figure 2*D* indicated significant differences between K4101 mutations and D4730N mutations, with *p* < 0.05 for all pairs by one-way ANOVA followed by Tukey’s test. This outcome implies that the impact of D4730 mutations on Ca^2+^ inhibition may exhibit an opposing trend compared to the effects of K4101 mutations in the EF-hand domain.

We also determined Ca^2+^ dependent activation of WT and mutant RyR1s. Although our hypothesis is that the domain interface between the EF hand and S2–S3 loop transmits inhibitory signaling, the K4101E mutation unexpectedly enhanced Ca^2+^-dependent activation of RyR1s (Fig. 2, *A* and *C*). A half maximal concentration of activating Ca^2+^ for the K4101E mutant is 0.13 ± 0.08 μM, which is significantly smaller than that of WT-RyR1, 0.46 ± 0.24 μM, while EC_50_ of activating Ca^2+^ for the other four mutants are essentially the same as WT-RyR1 (Fig. 2*C*).

### Magnesium-dependent inhibition of mutant RyR1s

**Figure 3:**
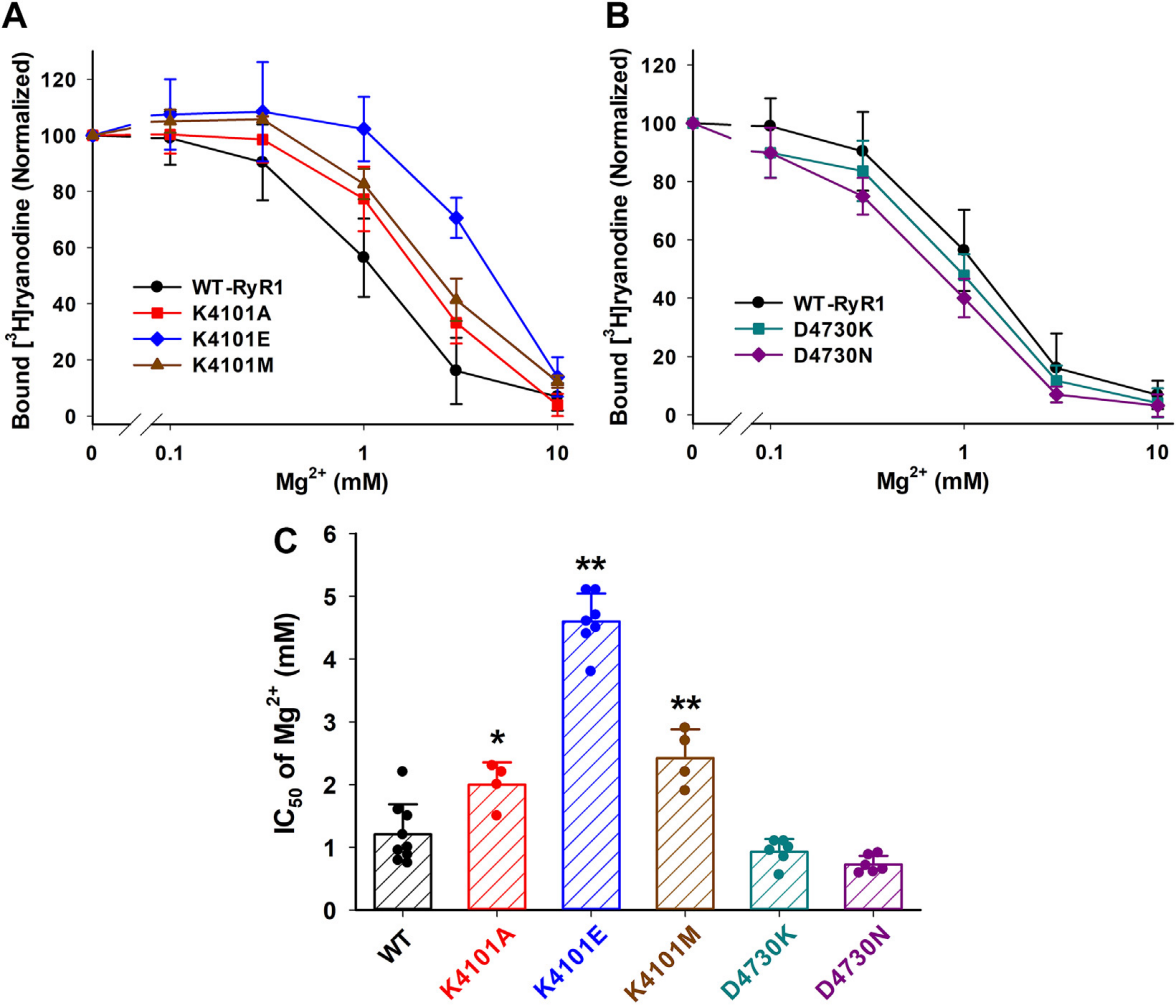
**Mg**^**2+**^**-dependent inhibitions of WT and mutant RyR1s.** Activities of WT and mutant RyR1s were determined in the presence of 165 μM Ca^2+^ and various concentrations of Mg^2+^ by [^3^H]ryanodine binding methods. *A* and *B*, Mg^2+^-dependent of bound [^3^H]ryanodine of K4101- and D4730-RyR1 mutants are shown together with WT-RyR1 in Panel *A* and *B*, respectively. *C*, half maximal Ca^2+^ concentrations for Mg^2+^ inhibition (IC_50_) are shown. Data are mean ± SD (n=4–9). ∗*p* < 0.05 and ∗∗*p* < 0.01 compared with WT-RyR1 by one-way ANOVA followed by Tukey’s test of six different genotypes of RyR1. RyR, ryanodine receptor.

Mg^2+^ is well-known to inhibit RyRs by at least two mechanisms: (a) Mg^2+^ binds to the high affinity Ca^2+^ activation site to prevent Ca^2+^ activation, and (b) Mg^2+^ binds to the low affinity Ca^2+^ binding site and works as an inhibitory divalent cation (13). In the second scheme, therefore, mutation effects on Ca^2+^ inhibition of RyR1 could correlate with the Mg^2+^-inhibitory effect. As shown in Figure 3*A*, mutations on K4101 significantly decreased Mg^2+^ inhibitory effects, which is consistent with impairment of Ca^2+^ inhibition. The IC_50_ of Mg^2+^ were 1.2 ± 0.5, 2.0 ± 0.4, 4.6 ± 0.4, and 2.4 ± 0.5 (in mM) for WT-, K4101A-, K4101E-, and K4101M-RyR1, respectively (Fig. 3*C*). D4730 mutations slightly decreased the mean IC_50_ values of Mg^2+^ (0.93 ± 0.20 and 0.73 ± 0.14 mM for D4730K and D4730 mutants, respectively), Figure 3, *B* and *C*. In consistent with Ca^2+^ inhibition (Fig. 2*D*), these values are not statistically different from that for WT-RyR1, but significantly smaller than those for K4101 mutants (*p* < 0.01 by one-way ANOVA followed by Tukey’s test). Taken together, the results suggest that Ca^2+^ and Mg^2+^ inhibition share a common intramolecular signaling pathway, which is mediated by interaction of the EF hand and S2–S3 domains through hydrogen bonds.

### Caffeine-dependent activation of mutant RyR1s

**Figure 4:**
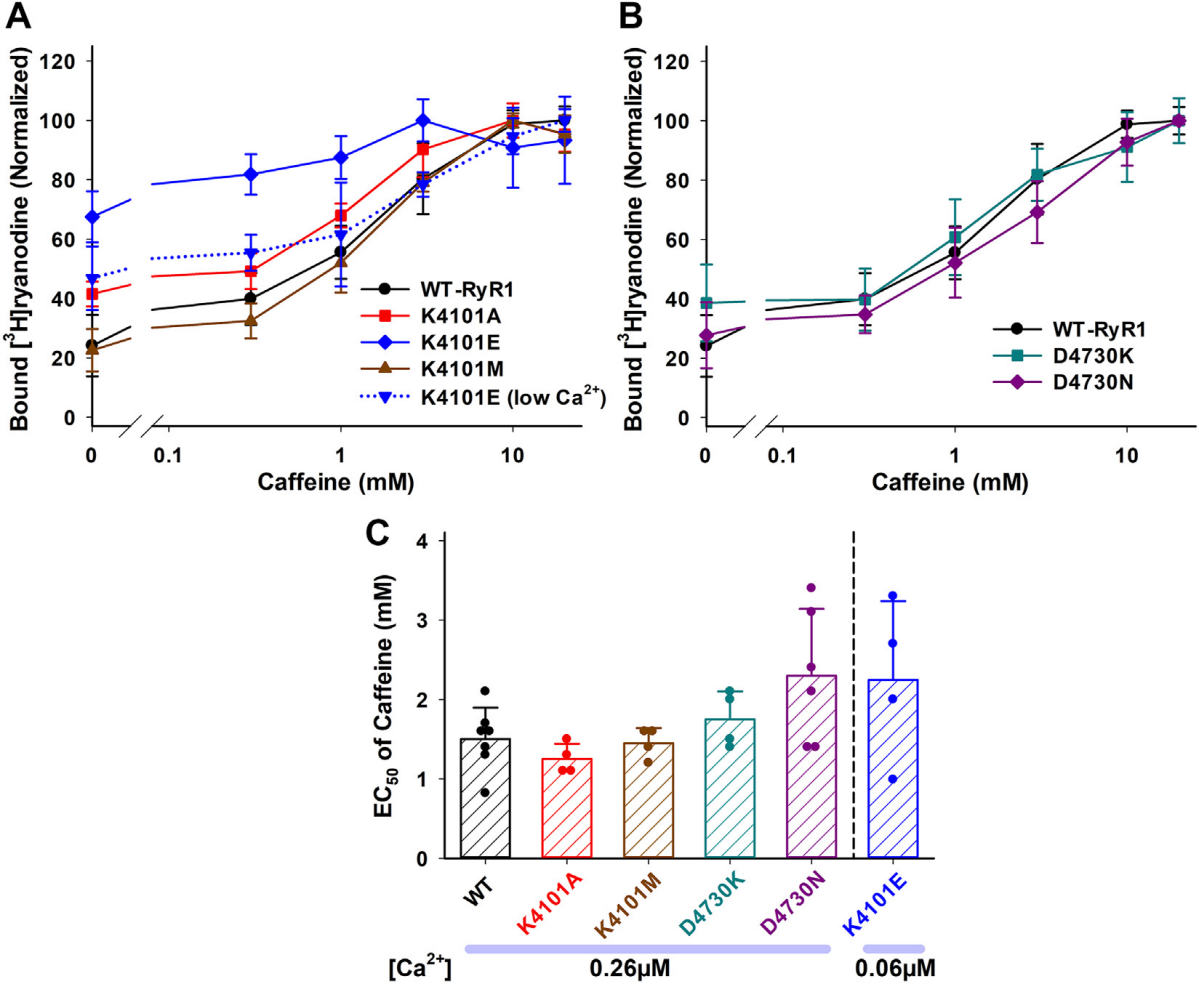
**Caffeine-dependent activations of WT and mutant RyR1s.** Activities of WT and mutant RyR1s were determined in the presence of 0.26 μM Ca^2+^ and various concentrations of caffeine by [^3^H]ryanodine binding methods. *A* and *B*, caffeine-dependent of bound [^3^H]ryanodine of K4101- and D4730-RyR1 mutants are shown together with WT-RyR1 in Panel *A* and *B*, respectively. Caffeine effect on K4101E mutant at 0.06 μM Ca^2+^ is shown as a *dotted blue line* in Panel *A*. Significant activations by caffeine in each genotype were determined by Kruskal-Wallis one-way ANOVA on Ranks followed by Dunn’s test for WT and K4101E or by one-way ANOVA followed by Tukey’s test for the other mutant RyR1s. All the mutant except K4101E at 0.26 μM Ca^2+^ (*solid blue line*) were significantly activated by 10 mM and 20 mM caffeine (*p* < 0.05). Significant activations of K4101E at 0.26 μM Ca^2+^ were only observed with 3 mM caffeine. *C*, half maximal Ca^2+^ concentrations for caffeine activation (EC_50_) are shown. Data are mean ± SD (n=4–7). EC_50_ values of the mutants (not including K4101E) were not significantly different from WT-RyR1 by either one-way ANOVA or Kruskal-Wallis one-way ANOVA on Ranks. RyR, ryanodine receptor.

Experiments with Ca^2+^- and Mg^2+^-dependent mutant RyR1 activity showed that the domain interface between the EF hand and S2–S3 loop mainly plays a role in RyR1 channel inhibitory mechanisms. We then examined the effect of another activating ligand, caffeine, on RyR1 domain interface mutants. As shown in Figure 4, *A* and *B*, at a subactivating Ca^2+^ concentration (0.26 μM) caffeine increased [^3^H]ryanodine binding to WT and mutant RyR1s by about 2.5- to 4-fold. The only exception to this effect is the K4101E mutant, because at 0.26 μM Ca^2+^ concentration, this mutant is already activated (Fig. 2). The EC_50_ values of caffeine were 1.5 ± 0.4, 1.3 ± 0.2, 1.5 ± 0.2, 1.8 ± 0.4, and 2.3 ± 0.8 (in mM) for WT-, K4101A-, K4101M, D4730K, and D4730N-RyR1, respectively (Fig. 4*C*). As seen in Figure 4*C*, the D4730N mutant showed variable EC_50_ values, which are not statistically significant but slightly larger than those of WT-RyR1. The effect of caffeine on the K4101E-RyR1 mutant was also determined at a lower Ca^2+^ concentration to obtain EC_50_ values. At 0.06 μM Ca^2+^, K4101E mutant was activated by caffeine in a comparable manner to the WT- and other mutant RyR1s at 0.26 μM Ca^2+^ (Fig. 4*A*). The EC_50_ of caffeine for K4101E mutant at 0.06 μM Ca^2+^ is 2.2 ± 1.0 mM (Fig. 4*C*).

### Calcium-dependent regulation of mutant RyR1s in the presence of caffeine or magnesium

We investigated Ca^2+^-dependent regulation of WT and selected mutant RyR1s in the presence of 3 mM caffeine or 0.5 mM Mg^2+^, concentrations proximal to the respective EC_50_ or IC_50_ values (Figs. 3*C* and 4*C*). Specifically, we focused on K4101E, K4101M, and D4730N mutants due to substantial changes observed in Ca^2+^/Mg^2+^ inhibition with K4101E and K4101M and contrasting effects displayed by D4730N compared to K4101 mutations (Figs. 2 and 3). In the presence of caffeine, the Ca^2+^ activation/inhibition profiles of WT and mutant RyR1s closely resembled those without caffeine. Fig. S1 demonstrated that K4101E and K4101M mutants adversely impaired Ca^2+^-dependent inhibition. D4730N mutation led to a decreased the mean IC_50_ value of Ca^2+^ as compared to WT-RyR1, although the difference was not statistically significant. In addition, we detected significant enhancement of Ca^2+^ activation in the K4101E mutant.

In the presence of 0.5 mM Mg^2+^, we observed less prominent effects by mutations (Fig. S2). K4101E significantly impaired Ca^2+^ inhibition; however, IC_50_ of Ca^2+^ for K4101M mutants remained essentially the same as that for WT-RyR1. A plausible explanation is that 0.5 mM Mg^2+^ is sufficiently high to inhibit the K4101M mutant; therefore, the mutation’s impact on Ca^2+^ inhibition, which shares in part a common signaling mechanism as Mg^2+^ inhibition (13), is minimized. On the other hand, the K4101E mutant is barely inhibited by 0.5 mM Mg^2+^ (Fig. 3*A*), and the mutation’s effects on both Ca^2+^ activation and inhibition remain significant.

### Computational analysis of hydrogen bond interactions in the EF hand and S2–S3 domain interface

**Figure 5:**
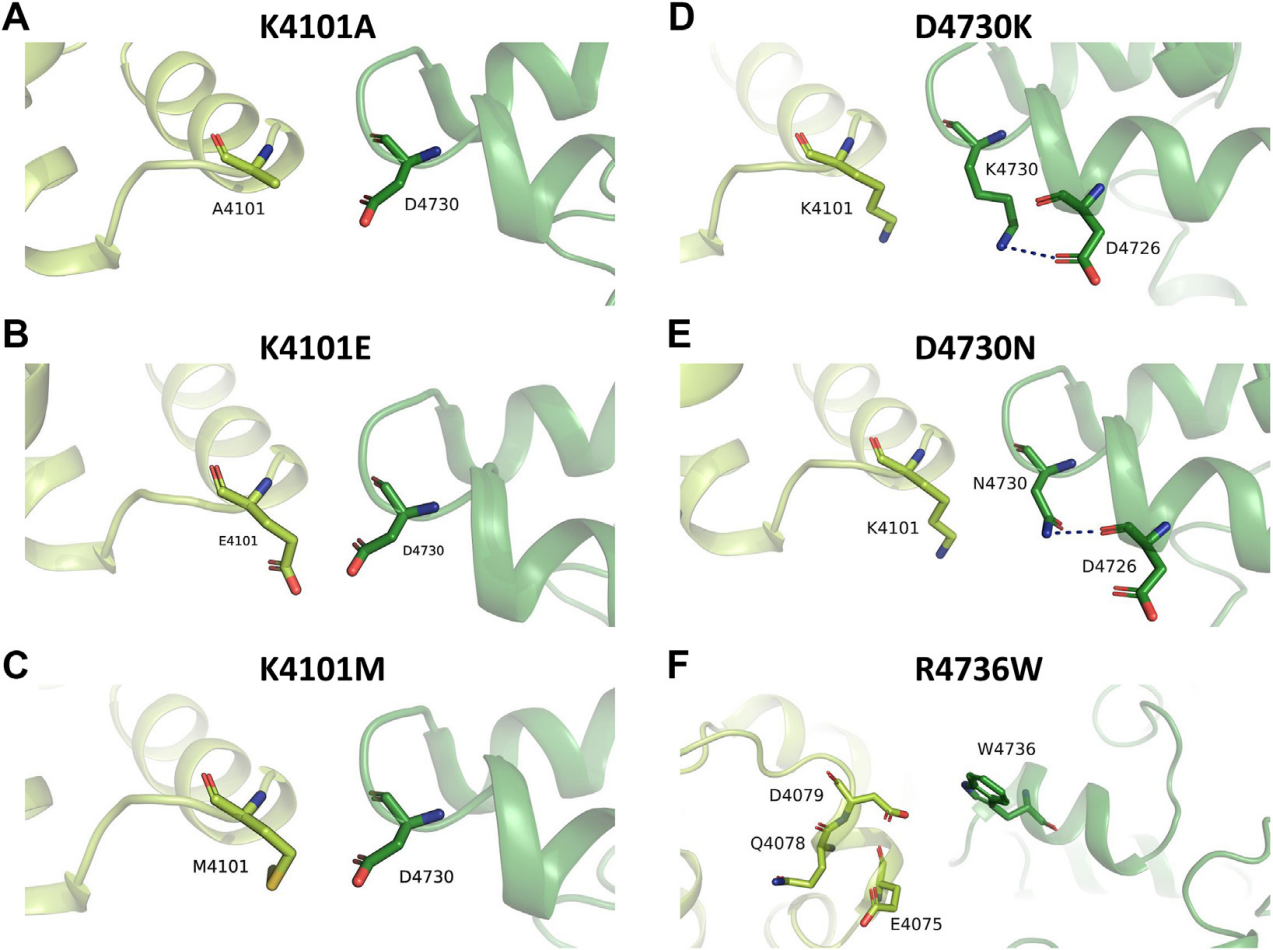
***In silico* mutagenesis of the interactions between EF and S2–S3 domains in RyR1**. PyMol was used to perform *in silico* mutagenesis based on the structure shown in Figure 1*B*. *A*–*F*, six different mutations on amino acids involved in hydrogen bond interactions (K4101, D4730, and R4736) were tested to observe changes in hydrogen bond interactions. The methods used for *in silico* mutagenesis are described in Experimental procedures. RyR, ryanodine receptor.

Here, we aimed to investigate the structural basis of impaired Ca^2+^ and Mg^2+^ inhibition in RyR1 mutants. To achieve this, we performed *in silico* mutagenesis of the EF hand and S2–S3 domain interface residues based on the structure with hydrogen bond interactions. Using this approach, we predicted the structural alterations induced by the mutations in all five RyR1 mutants generated for the current investigation (K4101A, K4101E, K4101M, D4730K, and D4730N) and the previously studied R4736W mutant (7), Figure 5. These mutant structures were then compared with the WT-RyR1 structure to discern any changes. Notably, our computational analyses revealed that all mutations reduced domain interactions, consistent with the functional outcomes observed in [^3^H]ryanodine binding assay for K4101 and R4736 mutants. However, the results with D4730 mutations were unexpected, as the mutations showed no change or even opposite functional effects to the K4101 mutations. It was interesting to note that D4730 mutations in the S2–S3 loop not only reduced the interaction with the EF hand domain but also induced a new interaction with D4726 in the same S2–S3 domain (Fig. 5, *D* and *E*). These findings suggest that the S2–S3 loop plays an important role in RyR1 regulation and that its mutations can have complex effects on protein function.

### Effect of the CPVT-associated mutation in RyR2 S2–S3 loop

**Figure 6:**
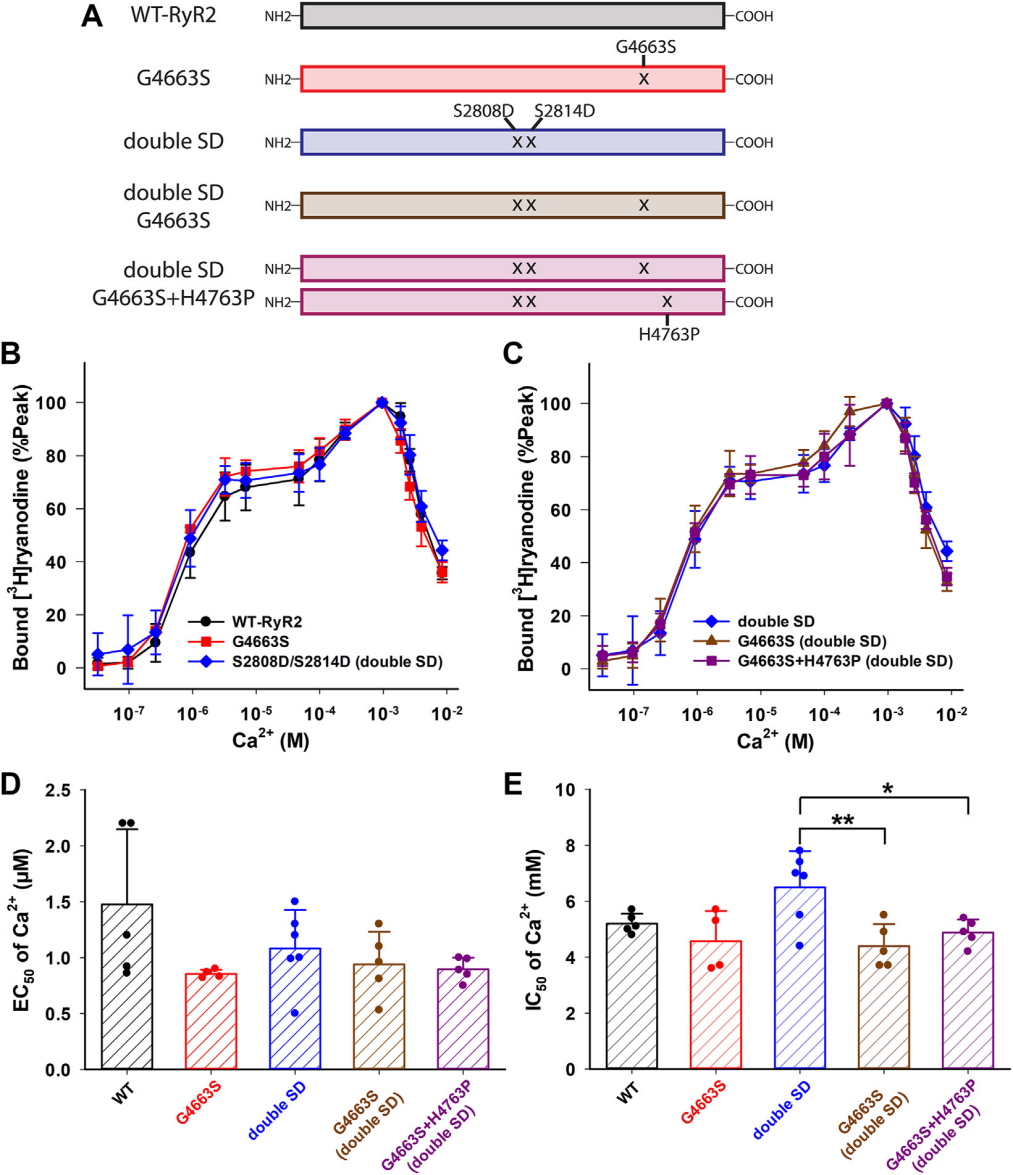
**Ca**^**2+**^-**dependent activity changes of WT and mutant RyR2s.***A*, schematic of WT and mutant RyR2s. *B* and *C*, activities of WT and mutant RyR2s were determined in the presence of various concentrations of Ca^2+^ by [^3^H]ryanodine binding methods. Ca^2+^ dependent of bound [^3^H]ryanodine of WT-, G4663S-, and double SD-RyR2 are compared in Panel *B*. G4663S-RyR2 and coexpression of G4663S- and H4763P-RyR2 at the phosphor-mimicking conformation are compared to double SD mutant in Panel *C*. *D* and *E*, half maximal Ca^2+^ concentrations for Ca^2+^ activation (EC_50_) and inhibition (IC_50_) are shown in Panel *D* and *E*, respectively. Data are mean ± SD (n=4–6). EC_50_ values of the mutants were not significantly different among the five groups by either one-way ANOVA or Kruskal-Wallis one-way ANOVA on Ranks. Significant differences of IC_50_ values among five groups were tested by one-way ANOVA followed by Tukey’s test. ∗*p* < 0.05 and ∗∗*p* < 0.01 compared with double SD-RyR2. RyR, ryanodine receptor.

We found that the domain interface between the EF hand and S2–S3 loop in RyR1 is crucial for Ca^2+^- and Mg^2+^-dependent inhibitory effects. In the previous report, two malignant hyperthermia-associated mutations, G4733E and R4736W in the S2–S3 loop particularly impaired RyR1 inhibition. While R4736 forms hydrogen bonds with EF hand amino acids (Fig. 1), G4733 is located at the domain interface. The RyR2-G4663S mutation, which is at the corresponding residue as the RyR1-G4733E, was reported to be associated with catecholaminergic polymorphic ventricular tachycardia (CPVT) (14). Onset of CPVT depends on catecholamine stimulation, and RyR2 phosphorylation at S2808 by PKA and/or S2814 by CaMKII are thought to correlate with arrhythmogenesis (15, 16). Interestingly, the CPVT patient with the G4663S-RyR2 mutation also carries a H4763P-RyR2 mutation on the other gene allele (14). Thus, in the present study we constructed four recombinant mutant RyR2s to investigate the effect of S2–S3 domain interface mutation (Fig. 6*A*): (a) G4663S-RyR2; (b) S2808D/S2814D-RyR2 (double SD), mimicking the phosphorylated conformation at the two serine residues; (c) double SD/G4663S-RyR2; and (d) coexpression of double SD/G4663S and double SD/H4763P. As shown in Figure 6, Ca^2+^-dependent activation and inhibition of these four mutant RyR2s are not different from those of WT-RyR2. However, Ca^2+^ inhibition of double SD/G4663S and coexpression of double SD/G4663S and double SD/H4763P were slightly enhanced as compared to the double SD mutant (Fig. 6*E*), maybe suggesting that G4663S and H4763P CPVT-associated mutations increase Ca^2+^-dependent inhibition when RyR2 is phosphorylated. Although these changes in IC_50_ of Ca^2+^ in G4663S or G4663S plus H4763P mutations at the phosphor-mimicking status are statistically significant, the differences are very subtle as compared to the mutation effect in RyR1s (K4101E in this study, and G4733E and R4736W in the previous study (7)). Thus, domain interface between the EF hand and S2–S3 loop may be less impactful for RyR2 channel inhibition by Ca^2+^.

## Discussion

### Calcium-dependent inhibition of RyRs

While molecular basis for Ca^2+^ activation of RyRs has been well-characterized in structural (11, 17) and functional (18, 19, 20, 21) experiments, little is known about the structure-function relationship of RyR inhibition by Ca^2+^. Inhibition of RyRs requires high Ca^2+^ concentrations, >100 μM Ca^2+^ for RyR1 and >1 mM for RyR2, which may be higher than physiological intracellular Ca^2+^ concentrations, particularly in cardiac muscle. Our present results showing contrasting effects by RyR1 and RyR2 domain interface mutations on Ca^2+^-inhibition (Figs. 2 and 5) raise the possibility that Ca^2+^-inhibition of RyR2 is not important at the normal conditions. Elevated cytosolic Ca^2+^ also inhibits both RyR1 and RyR2 through Ca^2+^ binding protein, calmodulin (CaM) and S100A1 (22, 23). Considering Ca^2+^ affinity of CaM (<10 μM), we propose that Ca^2+^/CaM-dependent RyR2 inhibition may be a more likely physiological mechanism rather than direct inhibition by Ca^2+^. Experiments with two genetically modified mice in which either RyR1 or RyR2 were not inhibited by Ca^2+^/CaM, showed a critical role for Ca^2+^/CaM inhibition of RyR2 but not RyR1 *in vivo* (24, 25), which aligns well with our current thoughts.

### Structural insights into the Ca^2+^ inhibition of RyR1

**Figure 7:**
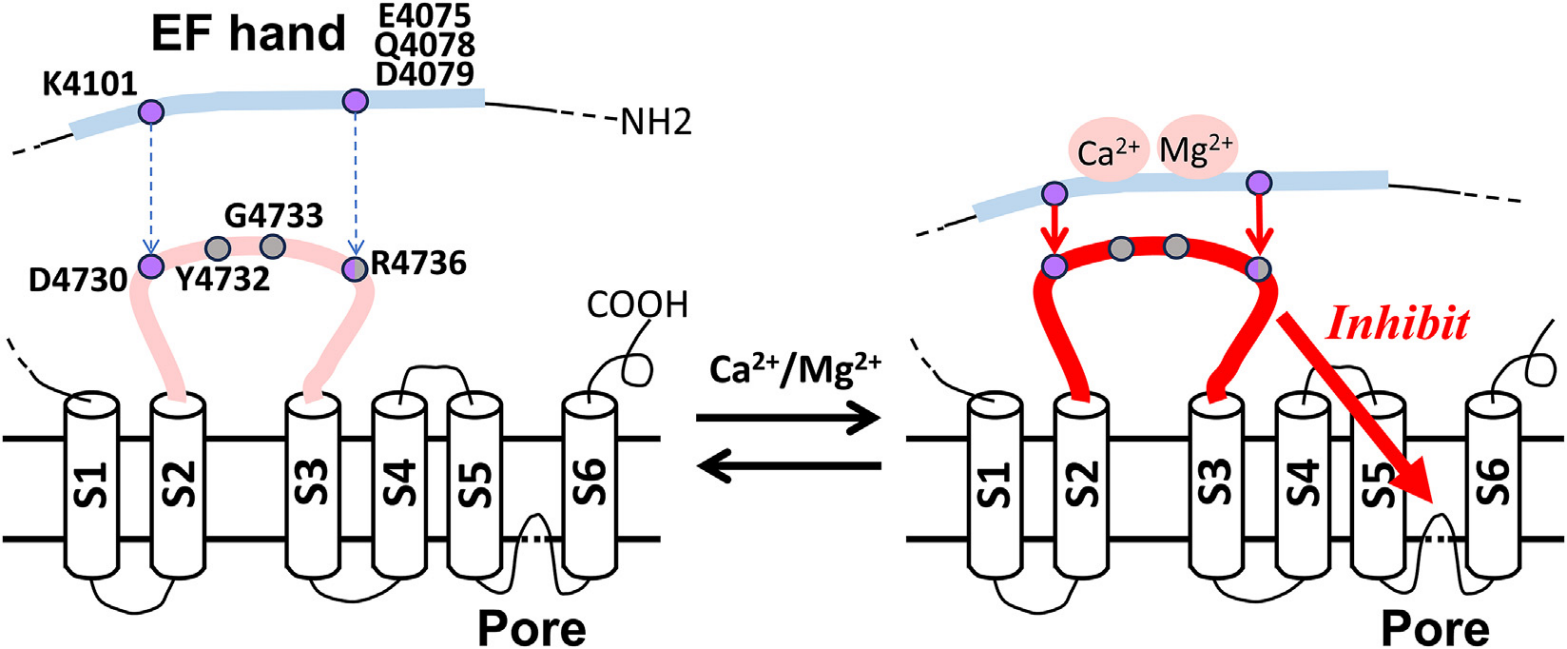
**Proposed model of Ca**^**2+**^**-dependent inhibition of RyR1.** Structural analysis revealed that a high concentration of Ca^2+^ leads to a reduction in the distance between EF hand and S2–S3 loop domains, resulting in the inhibited state of RyR1. Our present study proposes that this mechanism includes two pairs of hydrogen bond interactions between the amino acids highlighted as *purple dots*. Skeletal disease-associated mutation sites on the domain interface are shown as *gray dots*. RyR, ryanodine receptor.

During preparing for our functional studies involving the recombinant mutant RyR1s, Nayak and Samso reported a new set of RyR1 cryo-EM structures at ∼4 Å resolutions (12). These structures, captured in the presence of 2 mM AMP-PCP and 2 mM Ca^2+^, revealed two distinct ion conducting pore diameters of approximately 15.8 Å and 10.7 Å. Authors classified these as open and Ca^2+^-inactivated conformations of RyR1, respectively. Intriguingly, the distances between the EF hand and S2–S3 domains exhibited dynamic changes across the closed (in the presence of EDTA/EGTA), open, and Ca^2+^-inactivated states, with the shortest distance observed in the inactivated state. Additionally, the study identified two salt bridge formations, involving K4101 and D4730, as well as E4075 and R4736, aligning with our molecular dynamics simulations of the open state of RyR1 (Fig. 1). In their cryo-EM analysis, the distance between E4075 and R4736 underwent more drastic changes than the other salt bridge during these conformational changes. This finding could provide an explanation for our functional observation that R4736 mutations in the previous study (7) appeared to be more disruptive than K4101 and D4730 mutations in the present study. Overall, our current mutagenesis studies incorporating both computational and experimental data align with the cryo-EM observations at the EF hand and S2–S3 domain interface (12). Figure 7 proposes a plausible model for Ca^2+^ inhibition of RyR1. Upon Ca^2+^ (and probably Mg^2+^) binding to the EF hand, the domain distance between the EF hand and S2–S3 loop shortens, facilitating effective signal transmission through the two pairs of hydrogen bonds. However, the mechanism of inhibitory signal transmission between the S2–S3 loop and ion channel pore remains unresolved. The S2–S3 loop is in close proximity to the carboxyl terminal domain, which directly interacts with the S6 pore helix (11). Thus, ongoing efforts are aimed at identifying the domain interface between the S2–S3 and carboxyl terminal domain, including potential hydrogen bond interactions, to gain deeper insights into the molecular details of RyR1 inhibition.

A noteworthy observation from the study by Nayak and Samso (12) is that despite observing the closed (inactivated) state of RyR1 with a smaller pore size in the presence of 2 mM Ca^2+^, the Ca^2+^ binding site was not observed in the EF hand domain. Instead, Ca^2+^ binding was observed in the ATP binding pocket. Moreover, their molecular dynamics simulations showed that Mg^2+^ also binds to the ATP binding pocket; however, the Mg^2+^-inhibited conformation of RyR1 is different from the Ca^2+^-inhibited conformation. In our functional studies, we were unable to validate these structural distinctions, as changes in Ca^2+^-inhibition by mutations correlated well with changes in Mg^2+^-inhibition (Figs. 2*D* and 3*C*). A potential future experiment could involve investigating whether mutations in the ATP binding pocket differently alter Ca^2+^ and Mg^2+^ inhibition.

### Insights from the computational analysis of RyR1 domain interface

The *in silico* mutagenesis analysis primarily focused on amino acids involved in the hydrogen bond between the EF hand and S2–S3 loop (Fig. 6). The predicted outcomes of this analysis indicated that mutations on K4101 in the EF hand and R4736 in the S2–S3 loop would likely eliminate the hydrogen bond interaction, which is consistent with [^3^H]ryanodine binding assay in Figures 2 and 3 as well as findings from the previous article (7). Unexpectedly, the analysis predicted that the D4730 mutations in the S2–S3 domain would eliminate the hydrogen bond, while the functional experiments (Figs. 2 and 3) demonstrated that the D4730 mutations did not significantly change Ca^2+^ and Mg^2+^-dependent inhibition of RyR1. It is crucial to consider that the replacement of D4730 with lysine or asparagine might introduce a new hydrogen bond interaction potentially with D4726. Thus, one speculative explanation is that the structural rearrangement in the S2–S3 region caused by D4730K or D4730N mutations could compensate for the altered Ca^2+^ and Mg^2+^ inhibition resulting from the disruption of the hydrogen bond between K4101 and D4730.

### Structural difference between RyR1 and RyR2

In contrast to RyR1, the cardiac-disease associated RyR2 mutation, located in the domain interface between the EF hand and S2–S3 loop, did not exhibit a large impact on Ca^2+^ inhibition of RyR2 (Fig. 6). This might be due to a considerable difference in inhibitory Ca^2+^ affinity between WT-RyR1 and WT-RyR2 (4, 5, 6). To examine whether this functional difference correlates with structural dissimilarities, we measured the distance between the EF hand and S2–S3 domain interfaces in eight recently published RyR2 structures (17). We then compared these distances with those in four RyR1 structures, including one used for molecular dynamics simulations in Figure 1 (PDB: 5TAL). Our focus was on two pairs of amino acid interactions: (a) K4101 and D4730 of RyR1, corresponding to K4057 and E4660 of RyR2, and (b) E4075 and R4736 of RyR1, corresponding to D4031 and R4666. Table S1 shows that regardless of the ion channel’s open or closed states, domain distances in RyR1 are shorter than those in RyR2. Although the different sample preparation methods may affect the structure of domain interfaces, our current analysis demonstrated that the EF hand and S2–S3 loop domains in RyR2 are not as closely positioned as their counterparts in RyR1. This suggests that inhibitory Ca^2+^ signaling might not be as effectively transmitted between these domains in RyR2.

### Pathological relevance

Among five amino acids involved in hydrogen bond interactions between the RyR1 EF hand and S2–S3 domains (Fig. 1*F*), R4736 was reported to be associated with malignant hyperthermia (26, 27). Two other malignant hyperthermia associated amino acids, Y4732 (phenylalanine in rabbit) and G4733 (28, 29), do not form hydrogen bonds but are located in the domain interface between the EF hand and S2–S3 loop (12), Figure 7. In the previous study, we found all three amino acid mutations impaired Ca^2+^ and Mg^2+^ inhibition of RyR1 (7); therefore, one possible explanation is that the mutant RyR1s are less suppressed at both resting and activating states, resulting in the enhanced Ca^2+^- and caffeine-dependent muscle contractions, which are often observed in biopsies from the malignant hyperthermia susceptible patients. While clinical relevance with K4101- and D4730-RyR1 mutations has not yet been identified, three variants of the corresponding RyR2 amino acids, K4056N, K4056R, and E4659G (E4660G in the rabbit and pig sequences), were reported in ClinVar (https://www.ncbi.nlm.nih.gov/clinvar/). Although the arrythmia-associated G4663S-RyR2 mutation on the domain interface did not show an impact on Ca^2+^-dependent inhibition (Fig. 6), these three mutations directly occurring at the potential hydrogen bonding sites may alter RyR2 inhibition by Ca^2+^ and Mg^2+^.

### Study limitations and potential future experiments

By combining computational modeling, molecular dynamics simulations, and a functional mutagenesis approach, our current studies gained functional insights into RyR1 intersubunit interaction, which was suggested to be crucial for Ca^2+^-dependent regulation by structural studies (8, 12). Our approaches with the recombinant protein expression and *in vitro* assay enable us to determine RyR1 activity states at various conditions, thereby detecting important changes in ligand affinities to RyR1; however, we notice some limitations with our strategies. HEK293 cells do not express all the components required for skeletal excitation-contraction coupling. Also, our experimental platform is limited to examine the mutation effects on posttranslation modification of RyR1, but such analysis is particularly important when the disease-associated mutations are studied (30). In this respect, use of physiologically relevant systems such as genetically modified mouse models harboring RyR1 mutations or mutant RyR1 expression in the RyR-KO skeletal muscle cells will further validate the significance of hydrogen bond interaction on the domain interface in RyR1 channel regulations.

## Experimental procedures

### Materials

[^3^H]Ryanodine was purchased from PerkinElmer, unlabeled ryanodine from MilliporeSigma, protease inhibitors from Thermo Fisher Scientific and MilliporeSigma, and human embryonic kidney (HEK) 293 cells from American Type Culture Collection. Full-length rabbit RyR2 complementary DNA (cDNA) was provided by Dr Junichi Nakai (Tohoku University, Japan).

### Construction of RyR cDNAs

Full-length rabbit RyR1 (31) and RyR2 (32) were cloned into the mammalian expression vectors pCMV5 and pCIneo, respectively. Single and multiple base changes were introduced by *Pfu*-turbo polymerase-based chain reaction, using Nco*I*-Sma*I* (12418–12538) fragment of RyR1 for K4101 mutations or Bsr*GI*-Cla*I* (14189–14443) fragment for D4730 mutations as templates, mutagenic oligonucleotides, and QuikChange Site-Directed Mutagenesis Kit (Agilent Technologies). The mutated DNA fragments amplified by PCR were confirmed by DNA sequencing and were cloned back to the original positions. Full-length RyR1 expression plasmids were constructed by ligating three RyR1 fragments (Cla*I*-Xho*I* (polylinker-6598), Xho*I*-Eco*RI* (6598–11767), Eco*RI*-Xba*I* (11767-polylinker) carrying mutations) and pCMV5 expression vector (Cla*I*/Xba*I* cloning sites). For RyR2 S2808D/S2814D mutants, Mun*I*-Xba*I* (7735–10065) fragments were used as a template for site-directed mutagenesis. G4663S- and H4763P-RyR2 mutations were introduced in Bgl*II*-Bgl*II* (12614–14522) cDNA fragment. Sequences and numbering are consistent with previous studies (32, 33).

### Expression of full-length RyR1s in HEK293 cells

WT and mutant RyRs were transiently expressed in HEK293 cells by cDNA transfection using FuGENE6 or FuGENE HD transfection reagent (Promega). Since HEK293 cells express RyR protein at background level, we can determine the recombinant WT and mutant RyR channel activities by the heterologous expression (19). HEK293 cells were maintained at 37 °C with 5% CO_2_ in high glucose Dulbecco’s modified eagle medium containing 10% fetal bovine serum and plated ∼24 h before transfection. For each 10 cm culture dish, 3.5 to 5 μg cDNA was used for transfection, and cells were washed and harvested in the presence of protease inhibitors 48 h after transfection. The harvested cells were resuspended and homogenized in 0.3 M sucrose, 150 mM KCl, 20 mM imidazole (pH7.0), 0.1 mM EGTA, and protease inhibitors using a mechanical homogenizer, followed by centrifugation for 45 min at 100,000*g* to obtain crude membrane fractions containing the expressed RyRs. For RyR1 samples, 1 mM GSSG was added to the homogenizing solutions to minimize calpain digestions of RyR1 proteins (34). Resultant pellets were resuspended with the same buffer solution shown above without EGTA and glutathione.

### [^3^H]ryanodine binding assay

A plant alkaloid, ryanodine, prefers to bind to the open state of RyR ion channel (11, 35, 36); therefore, the radio-labeled [^3^H]ryanodine is widely used as a probe for RyR ion channel open/close activity, because the bound amount of [^3^H]ryanodine at equilibrium condition corresponds to the RyR channel open probability. [^3^H]Ryanodine binding experiments were performed with crude membrane fractions prepared from the HEK293 cells expressing recombinant RyRs to determine modulations of WT and mutant RyRs by Ca^2+^, Mg^2+^, and caffeine as described previously (6, 7). Membranes containing the recombinant RyRs were incubated with 3 nM [^3^H]ryanodine in 20 mM Hepes (pH 7.4), 0.15 M sucrose, 200 mM KCl, protease inhibitors, and various concentrations of Ca^2+^, Mg^2+^, and caffeine. Nonspecific and background binding of [^3^H]ryanodine was determined in the presence of ∼1000-fold excess amounts of unlabeled ryanodine. After 20 h, samples were diluted with about six volumes of ice-cold water and placed on Whatman GF/B filters preincubated with 2% polyethyleneimine in water. Filters were washed three times with 5 ml ice-cold solution containing 100 mM KCl and 1 mM K-Pipes (pH 7.0). The radioactivity remaining on the filters was determined by liquid scintillation counting to obtain bound [^3^H]ryanodine.

For each RyR1 sample, maximal binding (B_max_) values of [^3^H]ryanodine were determined to obtain RyR1 expression levels in HEK293 cells. The membranes were incubated with a nearly saturating 20 nM [^3^H]ryanodine for 4 h in 20 mM imidazole (pH 7.0), 0.6 M KCl, protease inhibitors, and 100 μM free Ca^2+^. Background binding of [^3^H]ryanodine was determined in the presence of 2.5 mM EGTA and 2.5 μM unlabeled ryanodine. The gain of activity for each RyR1 preparation was calculated by normalizing the peak values of Ca^2+^-dependent activity to B_max_ values. All experiments were performed at room temperature (23–25 °C).

### Free Ca^2+^ concentrations

Stock solutions (2×) with different free Ca^2+^ concentrations were prepared by including 300 mM KCl, 10 mM EGTA, and the appropriate amounts of CaCl_2_ in the absence of Mg^2+^ and ATP (pH 7.4 with 40 mM Hepes), as previously described (7). Final concentrations of KCl, EGTA, and Hepes are 150, 5, and 20 mM, respectively. To obtain a wide range of free Ca^2+^ concentrations, we first estimated free Ca^2+^ concentrations in the solution using the stability constants and the computer program published by Schoenmakers *et al.* (37) and Maxchelator (https://somapp.ucdmc.ucdavis.edu/pharmacology/bers/maxchelator/). Free Ca^2+^ concentrations in the stock solutions were measured with a Ca^2+^ selective electrode using a standard curve made with the Calcium Calibration Buffer Kit (Biotium) (<100 μM) and 1 M CaCl_2_ solution stock (Sigma-Aldrich) (≥100 μM). Although the measured free Ca^2+^ concentrations were not very different from the estimated ones by computer program, we used the measured values throughout the article. We prepared each Ca^2+^/EGTA buffer stock solutions in bulk and used them for all [^3^H]ryanodine binding experiments throughout this article for consistent comparative analysis.

### Data analysis

Ca^2+^-dependent activities of RyRs were normalized to the peak activity of each Ca^2+^-dependent bell-shaped curve, and half maximal concentrations of Ca^2+^ for activation and inhibition were determined as EC_50_ and IC_50_, respectively. Mg^2+^-dependent activities of RyR1s were determined in the presence of 165 μM free Ca^2+^ and various concentrations of Mg^2+^ and were normalized to the RyR1 activities without Mg^2+^ in each experiment. Caffeine-dependent activities of RyR1s were normalized to the maximal activities of caffeine-dependent activation curves in each experiment (typically activities with 10 mM or 20 mM caffeine).

Statistical analyses of data were performed by SigmaPlot 12 (Systat Software, https://systatsoftware.com/sigmaplot/). Results are given as means ± SD. Significant differences (*p* < 0.05) in the data of EC_50_, IC_50_, and gain were determined using one-way ANOVA followed by Tukey’s test. When data did not show normal distribution (by Shapiro-Wilk test) and/or variances of data were considered unequal, significant differences were also tested by Kruskal-Wallis one-way ANOVA on Ranks followed by Dunn’s test.

### Computational methods

We utilized the Ca^2+^-, ATP-, and caffeine-bound RyR1 structure in an open conformation (PDB: 5TAL) (11) as the starting point to simulate the dynamics of RyR1 as described previously (38). Briefly, we used the SWISS-MODEL tool (https://swissmodel.expasy.org/) (39) to replace the long missing segments in cryo-EM by short Gly loops. Using the CHARMM-GUI tool (40, 41) the transmembrane domain regions of modeled structures were embedded in 1-palmitoyl-2-oleoyl-glycero-3-phosphocholine (POPC)-lipid bilayers with dimensions 217 Å × 217 Å × 37 Å. The CHARMM36 force field (42, 43, 44, 45) was used on protein, membrane lipids, ATP, and caffeine molecules using GROMACS-2020 (https://www.gromacs.org/) (46). We explicitly solvated the membrane-inserted RyR1 system using the TIP3P water model, and the solvated systems were optimized. The equilibration simulations were performed for 10 ns with a time step of 2 fs, and the simulations were performed for 100 ns with a time step of 2 fs using GROMACS-2020 package (46). The 3D structural visualization of average RyR1 conformations extracted from respective trajectories was rendered using the PyMOL Molecular Graphics System (The Pymol Molecular Graphics System, Version 2.0. Schrödinger, LLC, New York, 2017, https://pymol.org/2/).

To evaluate hydrogen bonds between the S2–S3 and EF hand domains of RyR1, we used the gmx hbond tool. The gmx hbond tool calculates the number of hydrogen bonds between specified groups of atoms over time. We defined the donor and acceptor atoms involved in hydrogen bonding between the S2–S3 and EF hand domains and set a distance cutoff of 0.35 nm and an angle cutoff of 30 degrees.

*In silico* mutagenesis was performed using PyMOL to investigate the effects of specific amino acid substitutions on the structure and function of RyR1. Two sets of mutations were introduced: K4101A, K4101E, and K4101M, representing EF hand mutations, and D4730K, D4730N, and R4736W representing S2–S3 loop mutations. Using PyMOL's mutagenesis tool, the desired amino acid substitutions were introduced at positions 4101, 4730, and 4736. The mutated structures were then subjected to energy minimization and optimization. This *in silico* mutagenesis approach using PyMOL provides valuable insights into the structural consequences of EF hand and S2–S3 loop mutations in the RyR1 protein, contributing to a better understanding of its structure-function relationships.

## Data availability

All data are contained in the manuscript.

## Supporting information

This article contains supporting information (11, 17).

## Conflict of interest

The authors declare that they have no conflicts of interest with the contents of this article.
