# Supplementary material for: Structural and functional interactions between the EF hand domain and S2–S3 loop in the type-1 ryanodine receptor ion channel
Source: J Biol Chem. 2023 Dec 28;300(2):105606. doi: 10.1016/j.jbc.2023.105606 (PMC10832476; doi:10.1016/j.jbc.2023.105606)
Supplement: Supplemental Table S1, and Figs. S1 and S2 [file mmc1.docx]

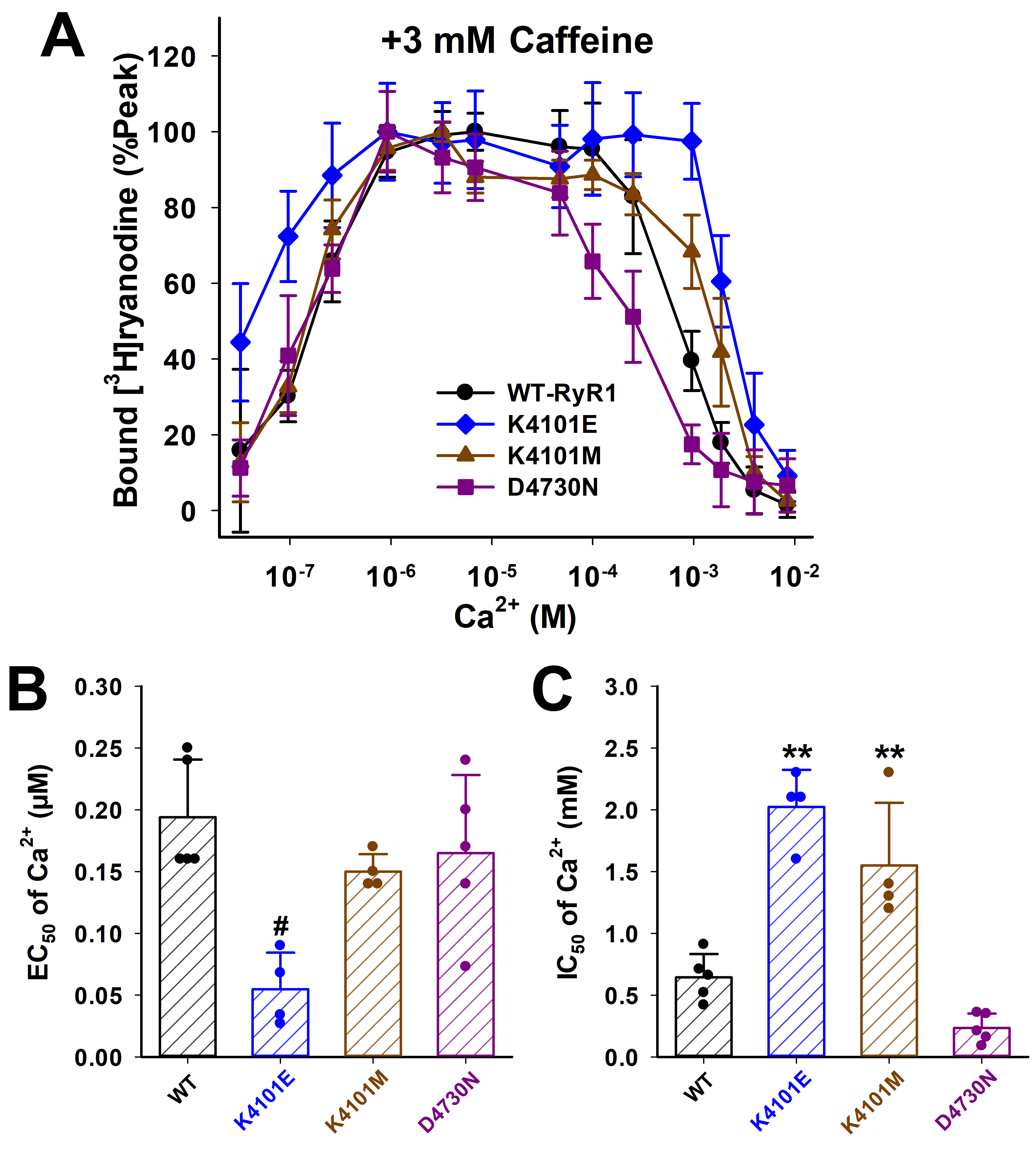
**Supporting Information**

**Supplementary Figure 1. Ca^2+^ dependent activity changes of wild type and mutant RyR1s in the presence of 3 mM caffeine.** Activities of WT and mutant RyR1s were determined in the presence of various concentration of Ca^2+^ and 3 mM caffeine by [^3^H]ryanodine binding methods. (A) Ca^2+^ dependent of bound [^3^H]ryanodine of K4101E-, K4101M-, and D4730N-RyR1 mutants are shown together with WT-RyR1. (B and C) Half maximal Ca^2+^ concentrations for Ca^2+^ activation (EC_50_) and inhibition (IC_50_) are shown in Panel B and C, respectively. Data are mean±SD (n=4-5). ^#^ p<0.05 compared with WT-RyR1 by one-way ANOVA on Rank followed by Dunn’s test of four different genotypes of RyR1. ** p<0.01 compared with WT-RyR1 by one-way ANOVA followed by Tukey’s test.


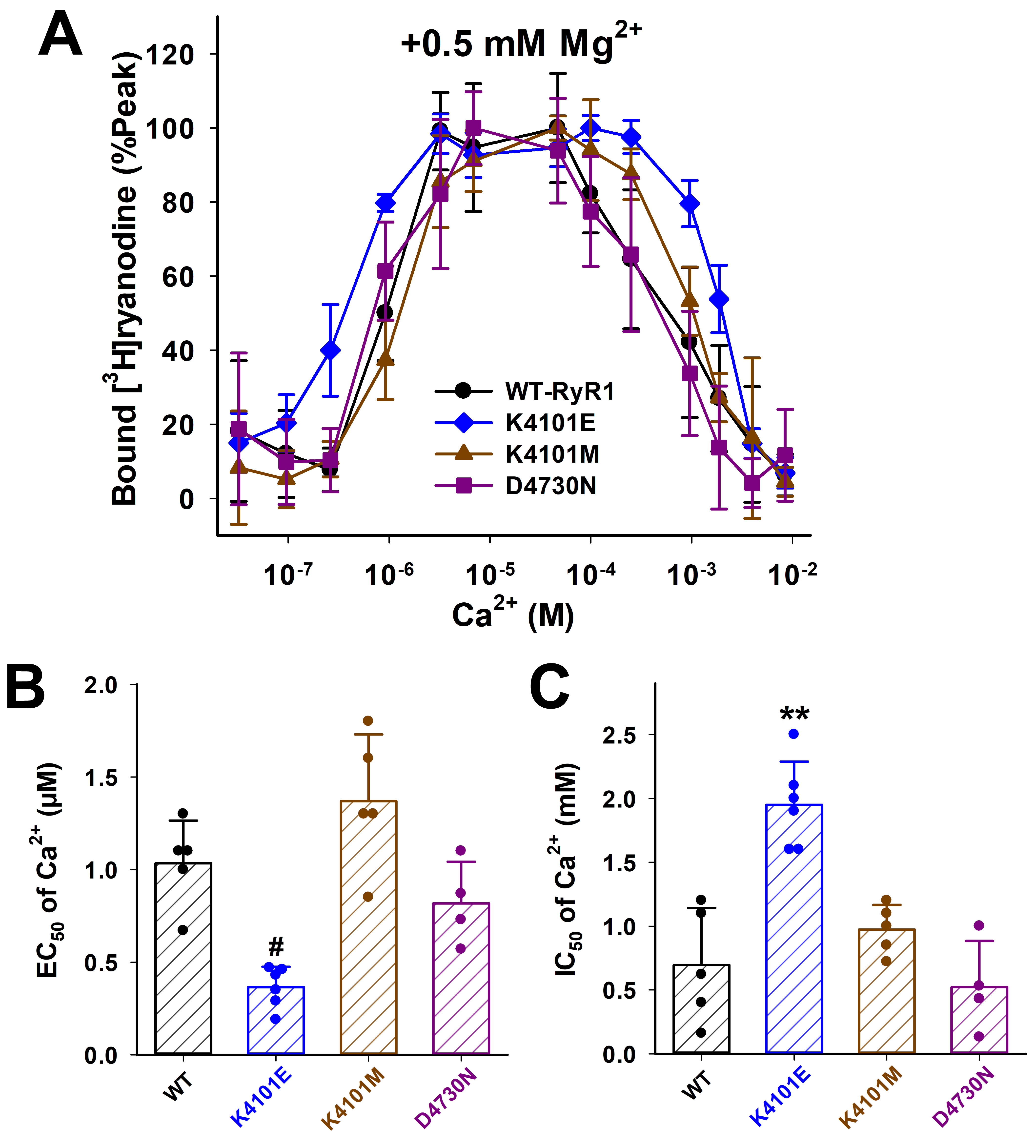


**Supplementary Figure 2. Ca^2+^ dependent activity changes of wild type and mutant RyR1s in the presence of 0.5 mM Mg^2+^.** Activities of WT and mutant RyR1s were determined in the presence of various concentration of Ca^2+^ and 0.5 mM Mg^2+^ by [^3^H]ryanodine binding methods. (A) Ca^2+^ dependent of bound [^3^H]ryanodine of K4101E-, K4101M-, and D4730N-RyR1 mutants are shown together with WT-RyR1. (B and C) Half maximal Ca^2+^ concentrations for Ca^2+^ activation (EC_50_) and inhibition (IC_50_) are shown in Panel B and C, respectively. Data are mean±SD (n=4-6). ^#^ p<0.05 compared with WT-RyR1 by one-way ANOVA on Rank followed by Dunn’s test of four different genotypes of RyR1. ** p<0.01 compared with WT-RyR1 by one-way ANOVA followed by Tukey’s test.

|  |  |  |  |  |  |
| --- | --- | --- | --- | --- | --- |
| RyR Structures | | | Distances (Å) | | |
| (PDB Number) | | | RyR1: K4101-D4730 |  | RyR1: E4075-R4736 |
|  |  |  | RyR2: K4057-E4660 |  | RyR2: D4031-R4666 |
| RyR1 | Open | 5TAL | 5.8 |  | 9.2 |
|  | Close | 5TAP | 5.8 |  | 8.0 |
|  |  | 5TAQ | 5.5 |  | 7.1 |
|  |  | 5T15 | 5.6 |  | 7.3 |
|  |  |  |  |  |  |
| RyR2 | Open | 6JII | 9.2 |  | 20.2 |
|  |  | 6JI0 | 11.4 |  | 20.9 |
|  |  | 6JIU | 9.6 |  | 21.8 |
|  |  | 6JRR | 9.6 |  | 18.8 |
|  |  | 6JRS | 8.0 |  | 20.2 |
|  |  | 6JIY | 9.4 |  | 21.5 |
|  | Close | 6JI8 | 9.4 |  | 17.6 |
|  |  | 6JV2 | 8.1 |  | 17.0 |
|  |  |  |  |  |  |

**Supplementary Table 1. Distance between EF hand and S2-S3 domain interface of RyR1 and RyR2.** Distances between the β-carbon of the indicated amino acid residues were determined using UCSF Chimera software (https://www.cgl.ucsf.edu/chimera/). RyR1 and RyR2 structures and sequences are from rabbit and pig (reference 11 and 17), respectively.
